# *Plasmodium malariae*—Repeat Light Microscopy when Molecular Testing is Not Available

**DOI:** 10.4269/ajtmh.18-0592

**Published:** 2019-02

**Authors:** Serena X. Zhang, Karl C. Kronmann, Michael J. Kavanaugh

**Affiliations:** 1 Department of Internal Medicine, Naval Medical Center Portsmouth, Portsmouth, Virginia;; 2 Department of Infectious Disease, Naval Medical Center Portsmouth, Portsmouth, Virginia

**Figure 1.**
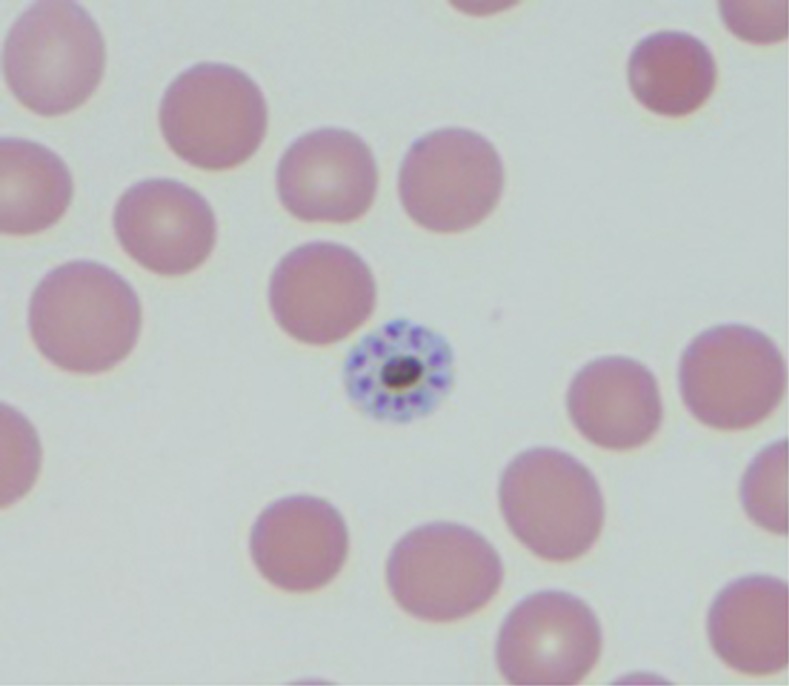
“Rosette” schizont. This figure appears in color at www.ajtmh.org.

**Figure 2.**
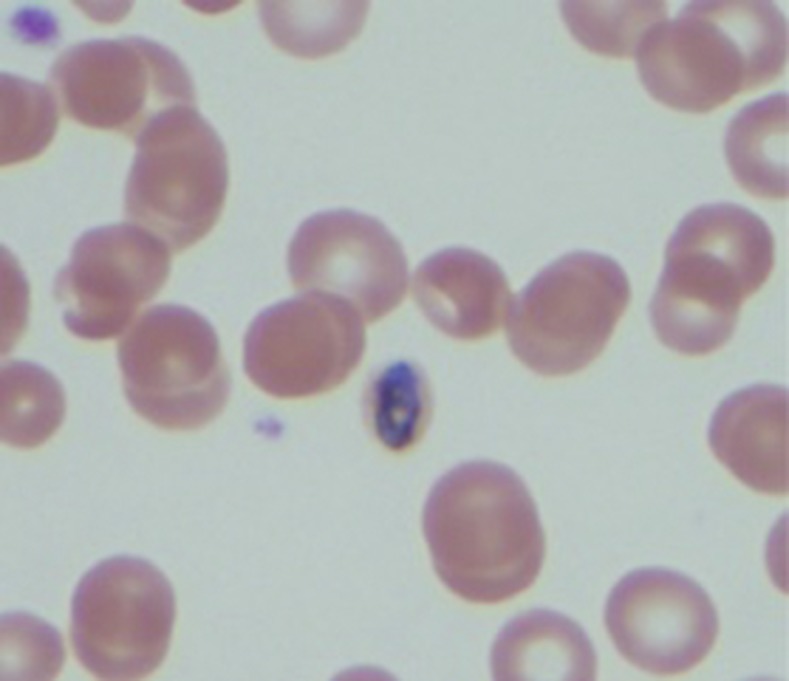
“Band”–developing gametocyte. This figure appears in color at www.ajtmh.org.

A 31-year-old previously healthy male required hospital admission after returning from rural Cameroon 10 weeks prior with severe myalgia, chills, 72 hours cyclical fever to 103.1°F, and tachycardia for 2 weeks. He endorsed adherence to atovoquone/proguanil chemoprophylaxis and recalled no exposure to lake or stream water. He was ill appearing, but without focal abnormalities. Laboratory findings were significant for leukopenia 2,900 cells/uL, thrombocytopenia 82,000 cells/uL, aspartate aminotranferase 316 units/L, and alanine aminotransferase 400 units/L. Initial three light microscopy (LM) and rapid diagnostic test (RDT) with BinaxNOW (Alere, Inc., Waltham, MA) were negative. However, continued investigation eventually revealed *Plasmodium malariae* on the fourth LM in its pathognomonic “rosette” schizont (Figure 1) and “band”–developing gametocyte (Figure 2).^1^ The patient was treated effectively with artemether/lumefantrine. *Plasmodium malariae* infections were once considered a rare and mild illness largely because of poor sensitivity on RDT and LM.^1–3^ However, recent improvement in polymerase chain reaction (PCR) technique increased the identification of *P. malariae* that might have been misdiagnosed as fever of unknown origin.^3–5^ Although there were reports of late onset and recrudescent fever despite adherent chemoprophylaxis, subsequent treatment has been paradoxically successful with the same medication.^3,5,6^ Because of *P. malariae*’s long senescent periods, recrudescent ability, and low parasite burden, clinicians must have high clinical suspicion and consider repeating LM when resource is limited or using PCR for diagnosis.^7^
